# Enteroviral Rhombencephalitis with Abducens Nerve Palsy and Cardio-Pulmonary Failure in a 2-Year-Old Boy

**DOI:** 10.3390/children9050643

**Published:** 2022-04-29

**Authors:** Chien-Yu Lin, Shih-Yu Huang, Chuen-Bin Jiang, Chun-Chih Peng, Hsin Chi, Nan-Chang Chiu

**Affiliations:** 1 Department of Pediatrics, Hsinchu MacKay Memorial Hospital, Hsinchu 30071, Taiwan; mmhped.lin@gmail.com; 2 Department of Medicine, MacKay Medical College, New Taipei 25160, Taiwan; ped.dr@yahoo.com.tw (C.-B.J.); pengcc4566@gmail.com (C.-C.P.); chi.4531@mmh.org.tw (H.C.); 3 Department of Internal Medicine, Hsinchu MacKay Memorial Hospital, Hsinchu 30071, Taiwan; 4174@mmh.org.tw; 4 Department of Pediatrics, MacKay Children’s Hospital, Taipei 10449, Taiwan

**Keywords:** enterovirus, rhombencephalitis, brainstem encephalitis, enterovirus infection with severe complications, intravenous immunoglobulin, extracorporeal membrane oxygenation

## Abstract

Enterovirus infection is endemic in many areas, especially in Southeast Asia. Enterovirus infection with severe complications (EVSC) is life-threatening, and timely diagnosis and management are crucial for successful management. Here, we report on a 2-year-old boy with hand, foot, and mouth disease. Myoclonic jerks developed and left abducens nerve palsy followed. Brain magnetic resonance imaging (MRI) showed rhombencephalitis. Pulmonary edema and cardiopulmonary failure developed, and intravenous immunoglobulin and extracorporeal membrane oxygenation were administered. He had a tracheostomy with home ventilator use after 64 days of hospitalization. At a 5-year follow-up, his neurodevelopment was normal with complete recovery from the abducens nerve palsy. The progress of EVSC may be rapid and fulminant, and timely diagnosis is critical for patient prognosis and outcomes. The presence of abducens nerve palsy is an indicator of enteroviral rhombencephalitis, and immediate and appropriate management is suggested.

## 1. Introduction

Enteroviruses, single positive-strand RNA viruses, belong to the genus Enterovirus in the family Picornaviridae. They comprise a group of 31 ubiquitous viruses, including coxsackieviruses, echoviruses, polioviruses, hepatitis A virus, enterovirus D68, and enterovirus A71 (EV71). Enteroviruses are transmitted by droplets, contact, and the fecal–oral route. Enterovirus infection is prevalent in many areas, and large endemics are not uncommon [1,2,3]. Enteroviruses can cause a variety of human diseases, and herpangina and hand, foot, and mouth disease (HFMD) are the most common forms. Most patients with enterovirus infection are mild and self-limited, but enterovirus infection with severe complications (EVSC) may be life-threatening [4]. There are five stages of EVSC, and stage-based management is associated with better patient outcomes [5]. The clinical course of EVSC is fulminant, and familiarity with the warning signs and stages of EVSC is crucial for successful patient treatment. Brainstem encephalitis (rhombencephalitis) caused by enterovirus is a lethal complication that results in extensive inflammation involving the hypothalamus, brainstem, spinal cord, and cerebellar dentate nucleus. It is characterized by the sudden onset of neurogenic pulmonary edema, and permanent neurologic sequela is common. Here, we report on a 2-year-old boy with HFMD and left abducens nerve palsy. Enteroviral rhombencephalitis was diagnosed.

## 2. Case Report

**Figure 1:**
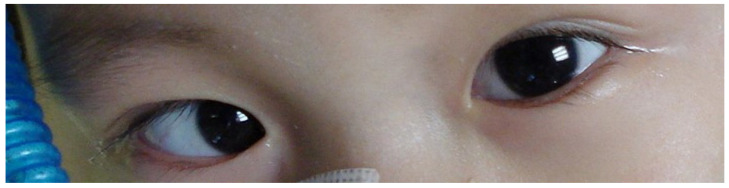
Eye movement was restricted in the left eye only on lateral gaze, and a left abducens nerve palsy was diagnosed.

**Figure 2:**
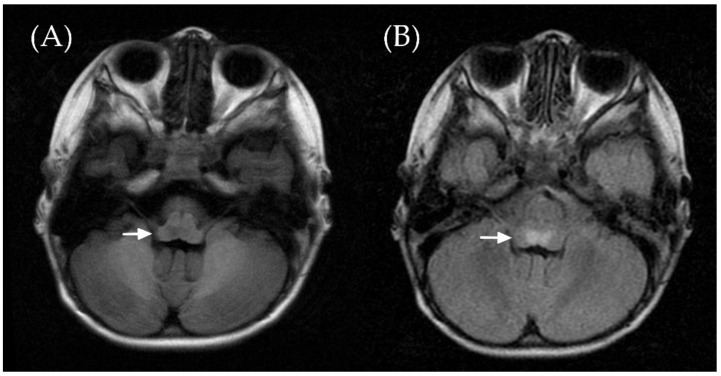
Brain magnetic resonance imaging showed symmetric lesions involving the dorsal pons and medulla oblongata, revealing brainstem encephalitis. These lesions (arrow) were hypointense in the T1-weighted FLAIR sequence panel (**A**) and hyperintense in the T2-weighted FLAIR images panel (**B**).

A 2-year-old boy had regular vaccination and was previously healthy. He presented with fever and myoclonic jerks of 2 days in duration. No cough, rhinorrhea, vomiting, or diarrhea were observed. On examination, multiple vesicles in the throat, hands, and feet were noted. Hand, foot, and mouth disease caused by an enterovirus was diagnosed, and he was admitted for dehydration and frequent myoclonic jerks. His consciousness was clear, and his neurologic examination was unremarkable. Blood tests showed mild leukocytosis (white blood cell count of 10,900 per microliter) and a normal C-reactive protein level. On the next day after hospitalization, eye movement was restricted in the left eye to lateral gaze (Figure 1). A spinal tap revealed pleocytosis (white blood cell count of 19 cells/µL with lymphocyte predominance). Brain magnetic resonance imaging showed symmetric lesions involving the dorsal pons and medulla oblongata, revealing rhombencephalitis (Figure 2). Brainstem encephalitis caused by enterovirus complicated with left abducens nerve palsy was diagnosed. Cardiopulmonary failure with pulmonary edema rapidly developed (Supplementary Figure S1), and intravenous immunoglobulin (IVIG) 1 g per kilogram for 1 dose and extracorporeal membrane oxygenation (ECMO) were administered. The nasopharyngeal swab polymerase chain reaction for EV71 was positive. The patient was discharged after 64 days of hospitalization. A tracheostomy with home ventilation was arranged, and he received rehabilitation and regular follow-up at our hospital. At a 5-year follow-up, his neurodevelopment was normal, with complete recovery from the abducens nerve palsy.

## 3. Discussion

Enterovirus infection remains an important health threat in many areas, and EVSC can be fatal. In 1998, a large endemic infection of EV71 occurred in Taiwan, and 405 children developed EVSC, resulting in 78 deaths [4]. Timely diagnosis and appropriate management are crucial for successful management, and our case showed the fulminant course of EV71 rhombencephalitis. Abducens nerve palsy may occur in EV71 rhombencephalitis, and aggressive monitoring and treatment should be initiated.

The abducens nerve is the sixth cranial nerve, and it is responsible for ipsilateral ocular abduction. It originates from the pons of the brainstem and travels into the skull base. It then passes through the cavernous sinus and enters the orbit. Diseases invading neurons or pathways will cause abducens nerve palsy, including trauma, vascular lesions, neoplasm, infection, and inflammation [6]. EV71 usually attacks the brainstem and may cause abducens nerve injury. Other viral encephalitis diseases, such as COVID-19, may also result in abducens nerve palsy [7]. EV71 encephalitis is a severe complication with high mortality and sequelae rates [8,9,10]. Long-term neurodevelopmental delay is also common, and timely management is critical to reducing sequelae. Our patient had cardiopulmonary failure, and he had to undergo a tracheostomy with home ventilator support. Fortunately, he had an uneventful recovery after aggressive rehabilitation.

In patients with EVSC, the direct invasion of viruses causes severe organ damage. Furthermore, alterations of cytokines may also play important roles in the pathophysiology of EVSC. Changes in several cytokines have been observed in patients with enterovirus encephalitis [11]. There is currently no available vaccine against enterovirus in Taiwan [12,13]. There is no specific antiviral agent against enterovirus infection, and most patients are treated with supportive care [14]. However, for critical patients with EVSC, early IVIG use may have immune-modulative effects and may improve clinical outcomes [15]. There are five stages of EVSC: (1) hand–foot–mouth disease/herpangina, (2) central nervous system (CNS) involvement, (3) autonomic nervous system dysregulation, (4) cardiopulmonary failure, and (5) convalescence. IVIG use is recommended for patients at stages 2 or 3, and ECMO treatment is recommended at stage 4. Compared to standard treatment, patients with stage-based management programs have been shown to have a significantly lower risk of EV71-related case fatality [5]. Familiarity with and the early recognition of each stage are beneficial for treating patients with EVSC. The presence of the abducens nerve is an important indicator of CNS involvement, and urgent management must be initiated.

Long-term neurologic sequelae and neurodevelopmental outcomes are major concerns in the survival of enteroviral encephalitis. The CNS involvement of EVSC may be divided into three groups: mild CNS involvement (aseptic meningitis), severe CNS involvement, and cardiopulmonary failure after CNS involvement [8,14], for which the recovery rates of neurologic development are 100%, 79%, and 25%, respectively [8]. Our patient had an uneventful recovery, and early IVIG treatment and ECMO use may play crucial roles in successful management. Although evidence supporting the benefits of IVIG treatment remains controversial, and the optimal dosage and timing of IVIG administration have not been established, it is safe for critical patients [16,17,18]. Furthermore, a high dose of IVIG (1 g/Kg) seems to be more effective than small doses [17]. Early IVIG use within 3 days of illness onset is associated with significantly higher survival rates in neonates with EVSC (52% with early IVIG vs. 13% without early IVIG) [18]. The progress of EVSC may be fulminant and rapid, and clinical conditions may deteriorate within hours. Familiarity with the warning signs of EVSC can contribute to the initiation of early IVIG use, intensive monitoring, inotropic agent and milrinone administration, ventilator support, and timely ECMO treatment.

## 4. Conclusions

Enterovirus infection is endemic in many areas, and EVSC is life-threatening. Disease progress is rapid, and timely diagnosis is crucial for successful management. Abducens nerve palsy may present in patients with EV71 rhombencephalitis, and an aggressive approach is recommended.

## Data Availability

Not applicable.

## Supplementary Materials

The following supporting information can be downloaded at: https://www.mdpi.com/article/10.3390/children9050643/s1, Figure S1: CXR revealed pulmonary edema on the following day.
