# Supplementary figures and images for: Enteroviral Rhombencephalitis with Abducens Nerve Palsy and Cardio-Pulmonary Failure in a 2-Year-Old Boy
Source: Children (Basel). 2022 Apr 29;9(5):643. doi: 10.3390/children9050643 (PMC9139552; doi:10.3390/children9050643)

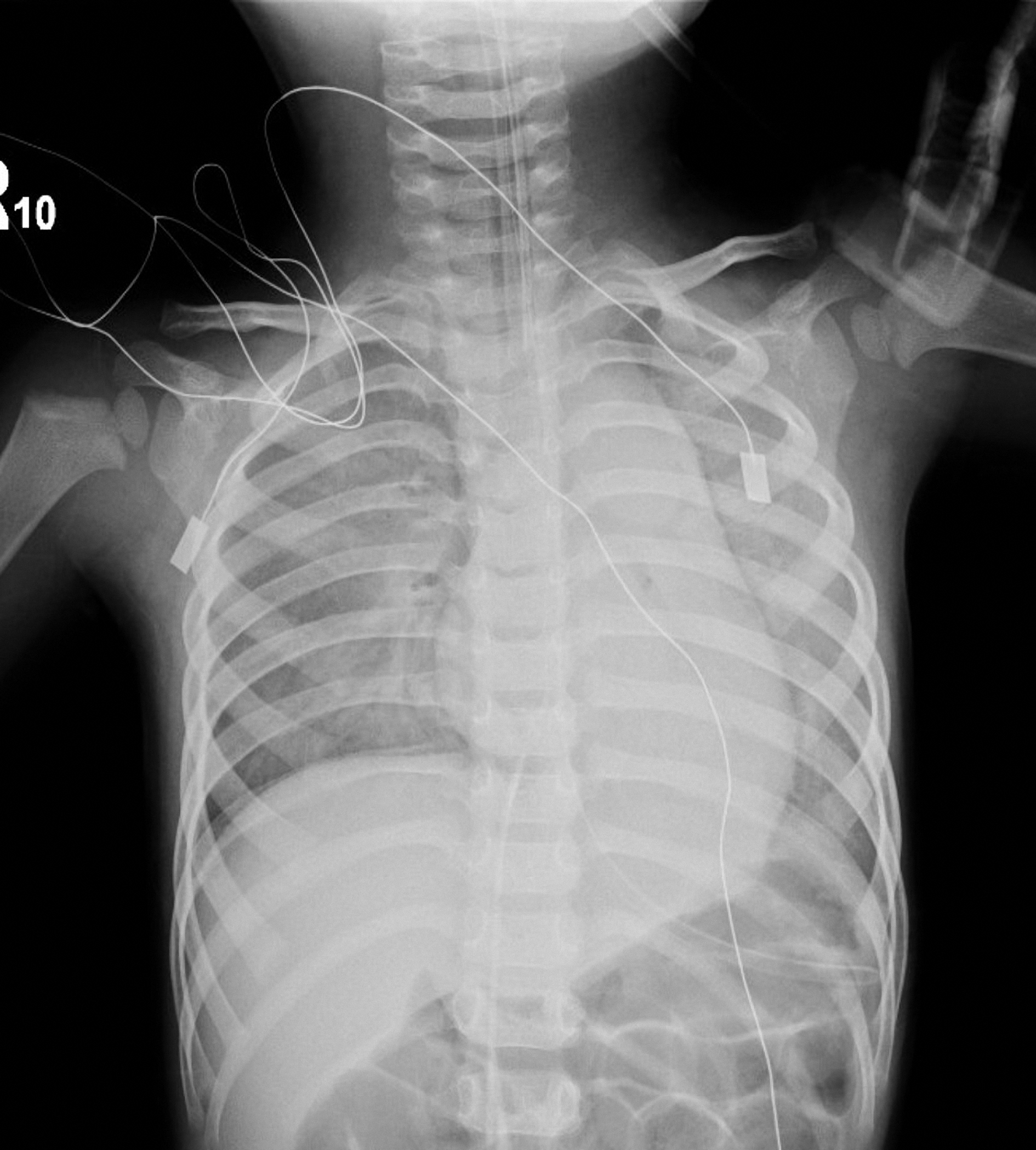

Supplement: Supplementary file 1 [file children-09-00643-s001.zip › Figure S1.jpg]
